# Combination of 12-*O*-tetradecanoylphorbol-13-acetate with diethyldithiocarbamate markedly inhibits pancreatic cancer cell growth in 3D culture and in immunodeficient mice

**DOI:** 10.3892/ijmm.2015.2163

**Published:** 2015-04-01

**Authors:** HUARONG HUANG, KAJIA CAO, SAQUIB MALIK, QIUYAN ZHANG, DONGLI LI, RICHARD CHANG, HUAQIAN WANG, WEIPING LIN, JEREMIAH VAN DOREN, KUN ZHANG, ZHIYUN DU, XI ZHENG

**Affiliations:** 1 Allan H. Conney Laboratory for Anticancer Research, Guangdong University of Technology, Guangzhou, Guangdong 510006, P.R. China; 2 Department of Nasopharyngeal Cancer, Sun Yat-sen University Cancer Center, Guangzhou, Guangdong 510060, P.R. China; 3 Department of Chemical Biology, Ernest Mario School of Pharmacy, Rutgers, The State University of New Jersey, Piscataway, NJ 08854, USA

**Keywords:** pancreatic cancer, 12-*O*-tetradecanoylphorbol-13-acetate, diethyldithiocarbamate, apoptosis, nuclear factor-κB

## Abstract

The aim of the present study was to determine the effects of 12-*O*-tetradecanoylphorbol-13-acetate (TPA) and diethyldithiocarbamate (DDTC) alone or in combination on human pancreatic cancer cells cultured *in vitro* and grown as xenograft tumors in nude mice. Pancreatic cancer cells were treated with either DDTC or TPA alone, or in combination and the number of viable cells was then determined by trypan blue ecxlusion assay and the number of apoptotic cells was determined by morphological assessment by staining the cells with propidium idiode and examining them under a fluorescence microscope. Treatment with DDTC or TPA alone inhibited the growth and promoted the apoptosis of pancreatic cancer cells in a concentration-dependent manner. These effects were more prominent following treatment with TPA in combination with DDTC than following treatment with either agent alone in PANC-1 cells in monolayer cultures and in 3 dimensional (3D) cultures. The potent effects of the combination treatment on PANC-1 cells were associated with the inhibition of nuclear factor-κB (NF-κB) activation and the decreased expression of Bcl-2 induced by DDTC, as shown by NF-κB-dependent reporter gene expression assay and western blot analysis. Furthermore, treatment of nude mice with DDTC + TPA strongly inhibited the growth of PANC-1 xenograft tumors. The results of the present study indicate that the administration of TPA and DDTC in combination may be an effective strategy for inhibiting the growth of pancreatic cancer.

## Introduction

Pancreatic cancer is one of the most aggressive types of cancer. It is estimated that this disease caused over 38,000 deaths in the United States in 2013 (1). Pancreatic cancer arises from the morphologically and genetically defined precursor lesions through a step-wise accumulation of genetic alterations. In the majority of patients diagnosed with the disease, symptoms do not develop until the tumor is either unresectable or metastatic, rendering it difficult to cure (2–4). Despite great advances in the treatment of cancer, pancreatic cancer is still the fourth most frequent cuase of cancer-related mortality in the Western world (1,2). The 5-year survival for individuals with pancreatic cancer is <5%, and conventional treatment approaches, such as surgery, radiation, chemotherapy, or combinations of these, have had little effect on the course of this aggressive neoplasm (3–7). The low survival rate of patients points towards an increased need for the development of novel anticancer agents and effective combination therapies for the treatment of pancreatic cancer.

**Figure 1:**
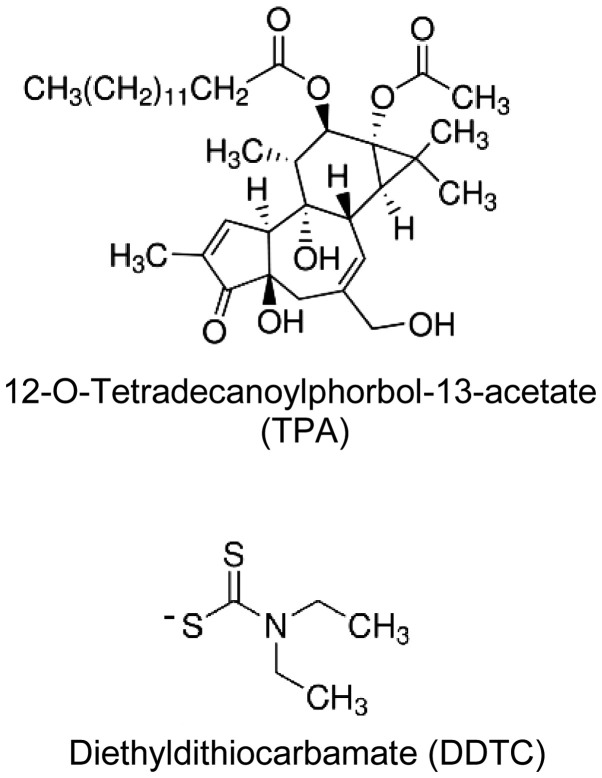
Structures of 12-*O*-tetradecanoylphorbol-13-acetate (TPA) and diethyldithiocarbamate (DDTC).

The phorbol ester, 12-*O*-tetradecanoylphorbol-13-acetate (TPA) (Fig. 1), is a major active constituent of the seed oil of *Croton tiglium* L., a leafy shrub of the Euphorbiaceae family that is native to Southeastern Asia. In previous studies by our group, we demonstrated the pharmacological activity of TPA in myeloid leukemia patients with an acceptable toxicity profile (8–10). Combinations with other agents have been shown to enhance the anticancer effects of TPA in myeloid leukemia and prostate cancer cells (11,12). Studies that have been carried out by our laboratory team, as well as other investigators have demonstrated that TPA inhibits the growth and induces the apoptosis of cultured pancreatic cancer cells (13–17). The nuclear factor-κB (NF-κB) transcription factor is constitutively activated in the majority of pancreatic cancers and is involved in the regulation of many aspects of tumor development and progression (18). Previous studies by our research team have indicated that the inhibition of NF-κB enhances the effects of TPA on leukemia and prostate cancer cells (19,20). Combination with pharmacological inhibitors of NF-κB may thus be an effective approach with which to increase the inhibitory effects of TPA on pancreatic cells.

Diethyldithiocarbamate (DDTC) (Fig. 1), a member of the dithiocarbamate family, is a potent inhibitor of NF-κB (21,22). DDTC is a major metabolite of disulfiram, an agent used in the treatment of alcoholism (23–25). Many clinical aspects of DDTC, such as the treatment of metal toxicity and cancer, have been investigated (26,27). Disulfiram, DDTC and pyrrolidine dithiocarbamate (PDTC) are well-known inhibitors of NF-κB. These compounds inhibit IκB phosphorylation, NF-κB nuclear translocation and proteasome degradation (21,22,28,29). DDTC has also been shown to induce the apoptosis of cancer cells (21,22,26). In addition, recent studies have demonstrated that a complex constituted by DDTC and copper inhibits the proliferation ofpancreatic cancer cells (30), and that DDTC synergistically enhances the effects of gemcitabine on pancreatic cells (31). Based on this evidence, we thus hypothesized that DDTC may inhibit the activation of NF-κB and may thus enhance the anticancer activity of TPA in pancreatic cancer cells.

The present study was undertaken to examine our hypothesis that DDTC enhances the growth inhibitory and apoptosis-promoting effects of TPA on pancreatic cancer cells. For this purpose, we determined the effects of DDTC and TPA alone or in combination on pancreatic cancer cells in conventional monolayer cultures, as well as in 3 dimensional (3D) cultures. In addition, the effects of TPA alone or in combination with DDTC on the growth of PANC-1 xenograft tumors in NCr nude mice were determined. To the best of our knowledge, the present study provides the first evidence that DDTC inhibits NF-κB activity, decreases the expression of Bcl-2 and enhances the inhibitory effects of TPA on pancreatic cancer cell growth *in vitro* and *in vivo*.

## Materials and methods

### Cells and reagents

The human pancreatic cancer cell lines, PANC-1, MIA PaCa-2 and BxPC-3, were obtained from the American Type Culture Collection (ATCC, Rockville, MD, USA). TPA was obtained from Alexis Co. (San Diego, CA, USA) and DDTC was from Sigma-Aldrich (St. Louis, MO, USA). The cells were maintained in Dulbecco’s modified Eagle’s medium (DMEM) containing 10% fetal bovine serum (FBS) that was supplemented with penicillin (100 U/ml)-streptomycin (100 *μ*g/ml) and L-glutamine (300 *μ*g/ml). The cultured cells were grown at 37°C in a humidified atmosphere of 5% CO_2_ and were passaged twice a week.

### Determination of the number of viable cells

The number of viable cells after each treatment was determined using a hemocytometer under a light microscope (Nikon Optiphot, Tokyo, Japan). Cell viability was determined by the trypan blue exclusion assay, which was performed by mixing 80 *μ*l of cell suspension and 20 *μ*l of 0.4% trypan blue solution for 2 min. Blue cells were counted as dead cells and the cells that did not absorb the dye were counted as live cells.

### Assessment of apoptotic cells by morphological analysis and by the activation of caspase-3

Apoptosis was determined by the morphological assessment of the cells stained with propidium iodide using a fluorescence microscope (Nikon Eclipse TE200; Nikon, Tokyo, Japan). Apoptotic cells were identified by classical morphological characteristics, including nuclear condensation, cell shrinkage and the formation of apoptotic bodies (20). The activation of caspase-3 was measured using an EnzoLyte AMC Caspase-3 Assay Fluorimetric kit (AnaSpec, Fremont, CA, USA) following the instructions of the manufacturer (32). Fluorescence intensity was measured using a Tecan Infinite M200 plate reader (Tecan US Inc., Durham, NC, USA).

### NF-κB-dependent reporter gene expression assay

NF-κB transcriptional activity was measured by NF-κB-luciferase reporter gene expression assay. The NF-κB-responsive luciferase construct was transiently transfected into the PANC-1 cells by using Lipofectamine™ 2000 (Invitrogen Life Technologies, Grand Island, NY, USA) following the manufacturer’s instructions. The cells were then treated with DDTC or TPA alone or in combination for 24 h, and the NF-κB-luciferase activities were measured using the luciferase assay kits (E1500; Promega Madison, WI, USA) according to the manufacturer’s instructions.

### Western blot analysis

Following treatment with TPA, DDTC or a combination of both, the cell lysates were prepared as described in a previous study (12). Proteins were subjected to sodium dodecyl sulfate-polyacrylamide gel electrophoresis (SDS-PAGE) and subsequently transferred onto nitrocellulose membranes. After blocking the non-specific binding sites with blocking buffer, the membranes were incubated overnight at 4°C with Bcl-2 primary antibody (05-729; Millipore Corp., Billerica, MA, USA). β-actin was used as a loading control. Following the removal of the primary antibody, the membranes were washed 3 times with TBS (PBS containing 0.05% Tween-20) buffer at room temperature and subsequently incubated with fluorochrome-conjugated secondary antibody (sc-3738; Santa Cruz Biotechnology, Inc., Santa Cruz, CA, USA). Final detection was performed using an Odyssey Infrared Imaging system (LI-COR Biosciences, Lincoln, NE, USA).

### 3D cell culture

The PANC-1 cells were mixed with Matrigel (Collaborative Research Inc., Bedford, MA, USA) on ice at a density of 0.5×10^5^ cells/ml. The Matrigel containing the PANC-1 cells was placed on a 12-well plate (1 ml/well) and incubated at 37°C for 2 h to allow the Matrigel to solidify. Subsequently, DMEM was added to each well on top of the gel. The cells were incubated for 24 h and then treated with DDTC or TPA alone or in combination once every other day. On day 10, the 3D cultures were examined under a microscope (Nikon Optiphot; Nikon) for the determination of the formation of tissue-like structures.

### Xenograft tumors in NCr nude mice

Male NCr nude mice (6–7 weeks old) were obtained from Taconic Farms Inc. (Germantown, NY, USA). The animals were housed in sterile filter-capped microisolator cages and provided with sterilized food and water. The PANC-1 cells (2×10^6^ cells/mouse) suspended in 50% Matrigel (Collaborative Research Inc.) in DMEM were injected subcutaneously into the right flank of the mice. When the tumors reached a moderate size (0.6–1.0 cm in width and 0.6–1.0 cm in length), the mice received daily intraperitoneal (i.p.) injections with solvent (controls) which consisted of propylene glycol, polysorbate 80, benzyl alcohol, ethanol and water (40:0.5:1:10:48.5; control), TPA (50 ng/g body weight/day), DDTC (30 *μ*g/g body weight/day) or a combination of TPA (50 ng/g/day) and DDTC (30 *μ*g/g/day) for 28 days. Tumor size (length × width) and body weight were measured 3 times a week. The animal experiments were carried out under an Institutional Animal Care and Use Committee (IACUC)-approved protocol (Rutgers University).

### Tumor cell proliferation

The proliferation of the PANC-1 tumor cells was determined by the expression of proliferating cell nuclear antigen (PCNA) using immunohistochemical staining. In brief, tumors were excised from each mouse and weighed at the end of the experiment. Tumor tissues were fixed in buffered formalin for 24 h and then with ethanol for 48 h. Paraffin blocks of tumor tissues were prepared and paraffin sections of tumor tissues were processed for immunohistochemical staining. The sections were incubated with PCNA antibody (MAB424; Millipore Corp.) for 1 h at room temperature. The sections were then incubated with a biotinylated secondary antibody for 30 min followed by incubation with horseradish peroxidase conjugated-avidin solution for 30 min using the Elite ABC kit (PK-6100; Vector Laboratories, Burlingame, CA, USA). PCNA staining in the tumor cells (brown color in nucleus) was examined under a microscope (Nikon Optiphot; Nikon). At least 1,000 cells were counted for each section.

### Statistical analysis

The analysis of variance (ANOVA) method and the Tukey-Kramer test were used for the comparison of viable cells, apoptosis and NF-κB luciferase activity in the cultured pancreatic cancer cells. These statistical methods were also used for the comparison of tumor size and body weight among the different treatment groups in the *in vivo* experiments. A P-value <0.05 was considered to indicate a statistically signficant difference.

## Results

### Effects of TPA and DDTC on the growth and apoptosis of pancreatic cancer cells

**Figure 2:**
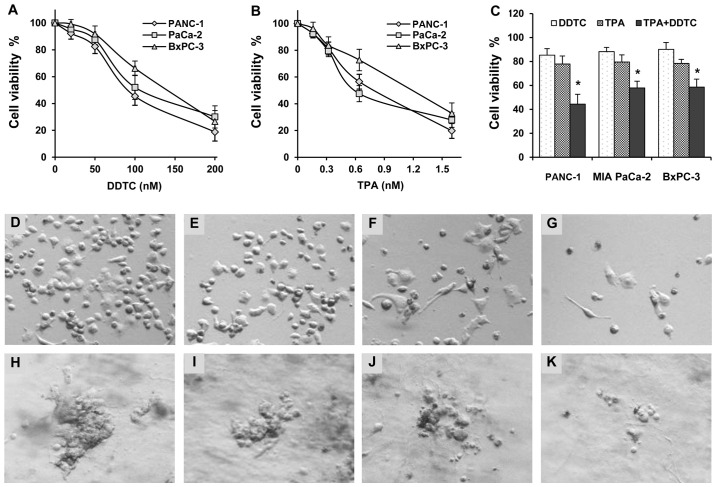
Effects of 12-*O*-tetradecanoylphorbol-13-acetate (TPA) and diethyldithiocarbamate (DDTC) alone or in combination on human pancreatic cancer cells. (A-C) PANC-1, MIA PaCa-2 and Bx-PC-3 cells were seeded at a density of 0.2×10^5^ cells/ml in cell culture dishes and incubated for 24 h. The cells were then treated with TPA or DDTC alone or in combination for 72 h. The number of viable cells was determined by the trypan blue exclusion assay. ^*^P<0.001, compared to treatment with either agent alone. (D-G) Morphology of PANC-1 cells treated with TPA or DDTC alone or in combination as described above. Representative micrographs of PANC-1 cells in the (D) control, (E) DDTC-treated, (F) TPA-treated and (G) TPA + DDTC-treated groups are shown. (H-K) Morphology of PANC-1 cells in 3D culture. PANC-1 cells were seeded at a density of 0.5×10^5^ cells/ml in Matrigel in a 12-well plate (1 ml/well) and incubated for 2 h to allow the gel to solidify. DMEM was added on top of the gel (1 ml/well), and the cells were incubated for 24 h. The cells were then treated with TPA and DDTC alone or in combination once every other day for 10 days. Representative micrographs of PANC-1 cell 3D cultures in the (H) control, (I) DDTC-treated, (J) TPA-treated and (K) TPA + DDTC-treated groups are shown.

The effects of TPA and DDTC alone or in combination on the growth of human pancreatic cancer cells were determined using the trypan blue exclusion assay. Treatment of the human prostate cancer cells, PANC-1, MIA PaCa-2 and BxPC-3, with TPA or DDTC alone resulted in cancer cell growth inhibition in a concentration-dependent manner (Fig. 2A and B). The PANC-1 cells were more sensitive than the MIA PaCa-2 and BxPC-3 cells to the growth inhibitory effects induced by TPA and DDTC (Fig. 2A and B). The combination of DDTC and TPA had more potent inhibitory effects on the growth of the cells than either agent alone (Fig. 2C). The number of viable PANC-1, MIA PaCa-2 and BxPC-3 cells was significantly lower in the group treated with the combination of both agents than in the groups treated with either TPA or DDTC alone (P<0.001). We also observed the morphology of the PANC-1 cells treated with TPA and/or DDTC under a phase-contrast microscope (Fig. 2D–G). The effects of TPA and/or DDTC on the apoptosis of the PANC-1 cells were determined by morphological assessment and caspase-3 assay. Treatment with TPA or DDTC alone resulted in a moderate increase in the number of apoptotic cells (Table I). The combination treatments with TPA and DDTC at various concentrations had a more potent promoting effect on apoptosis than treatment with either agent alone (Table I).

### Effects of TPA and DDTC on PANC-1 cells in 3D culture

A 3D cell culture model was used to determine the effects of TPA and DDTC alone or in combination on the formation and growth of 3D tissue-like structures. The PANC-1 cells formed a tissue-like morphology in 3D culture in the extracellular matrix gel (Fig. 2H). Treatment with DDTC or TPA alone had an inhibitory effect on the formation and growth of tissue-like structures (Fig. 2I and J). DDTC and TPA in combination had a more potent inhibitory effect on the formation of tissue-like structures (Fig. 2K).

### Effects of TPA and/or DDTC on NF-κB activation and the expression of Bcl-2

**Figure 3:**
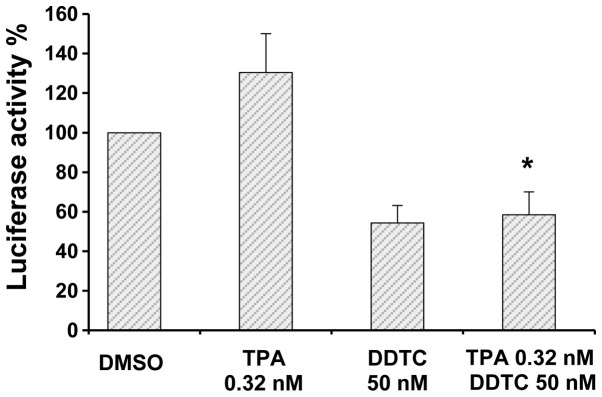
Effects of 12-*O*-tetradecanoylphorbol-13-acetate (TPA) and/or dieth-yldithiocarbamate (DDTC) on nuclear factor-κB (NF-κB) activity. PANC-1 cells were seeded at a density of 0.2×10^6^ cells/ml in medium in 60 mm culture dishes (5 ml/dish) and incubated for 24 h. The cells were then transfected with a NF-κB-luciferase construct using Lipofectamine™ 2000 (LF2000; Invitrogen Life Technologies). The cells were treated with TPA alone or in combination with DDTC for 24 h. The luciferase activity was determined using a lucif-erase assay kits (E1500; Promega). Each value represents the mean ± SE from 3 separate experiments. ^*^P<0.01 compared to treatment with TPA alone.

**Figure 4:**
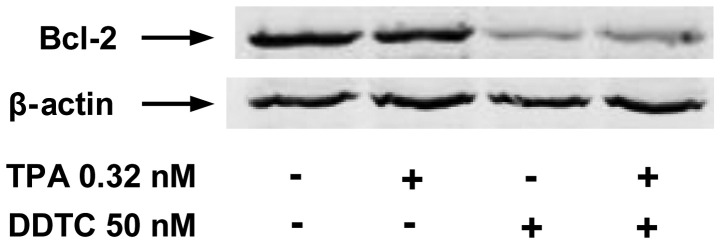
Effect of 12-*O*-tetradecanoylphorbol-13-acetate (TPA) and/or dieth-yldithiocarbamate (DDTC) on the level of Bcl-2 in PANC-1 cells. PANC-1 cells were seeded at a density of 1×10^5^ cells/ml in medium and incubated for 24 h. The cells were then treated with TPA or DDTC alone or in combination for 48 h. Bcl-2 expression was measured by western blot analysis using an anti-Bcl-2 antibody (05-729; Millipore Corp.).

The effects of TPA and DDTC alone or in combination on the activation of NF-κB were determined by luciferase reporter gene expression assay. Treatment of the PANC-1 cells with DDTC resulted in a marked decrease in NF-κB activity, while treatment with TPA alone caused an increase in the activity of NF-κB (Fig. 3). The stimulatory effects of TPA on NF-κB were markedly suppressed by treatment with DDTC (combination treatment; Fig. 3). The expression of Bcl-2, a downstream target of the NF-κB pathway was measured by western blot analysis. Treatment with TPA alone had little or no effect on the level of Bcl-2 (Fig. 4). However, treatment of the PANC-1 cells with DDTC alone or in combination with TPA resulted in a marked decrease in the level of Bcl-2 (Fig. 4).

### Inhibitory effect of TPA or DDTC alone or in combination on the growth of PANC-1 xenograft tumors in NCr nude mice

**Figure 5:**
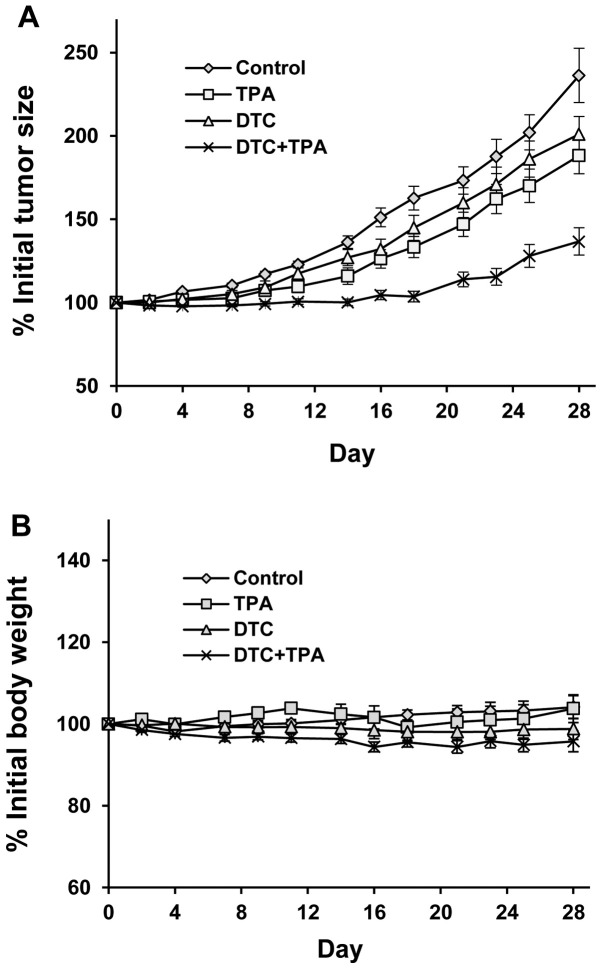
Effects of intraperitoneal injections of 12-*O*-tetradecanoylphorbol-13-acetate (TPA) or diethyldithiocarbamate (DDTC) alone or in combination on (A) the growth of PANC-1 tumors and (B) the body weight of NCr nude mice. Male NCr nude mice were injected subcutaneously with PANC-1 cells (2.0×10^6^ cells/mouse). Mice with established tumors (0.6-1.0 cm wide and 0.6-1.0 cm long) received daily intraperitoneal injections of TPA or DDTC alone or in combination for 28 days. Tumor size (length × width) and body weight were measured and expressed as a percentage of the initial tumor size or a percentage of the initial body weight. Each value represents the mean ± SE.

NCr nude mice bearing PANC-1 xenograft tumors were treated with daily an i.p. injection of TPA or DDTC alone or a combination of both for 28 days. Tumor growth was also observed in the control group (Fig. 5A). Treatment with i.p. injections of TPA in combination with DDTC had a more prominent inhibitory effect on the growth of PANC-1 tumors than either agent used individually (Fig. 5A). Statistical analysis using ANOVA with the Tukey-Kramer multiple comparison test revealed that the differences in the percentage of the initial tumor size at the end of the experiment were statistically significant between the control group and the group treated with the combination of both agents (P<0.001), as well as between the control group and the TPA-treated group (P<0.05). The percentage of the initial tumor size in the group treated with the combination of both agents was significantly smaller than that in the groups treated with TPA alone (P<0.05) or DDTC alone (P<0.01). Treatment with TPA or DDTC alone or in combination did not significantly affect the body weight of the animals (Fig. 5B). Statistical analysis using ANOVA with the Tukey-Kramer multiple comparison test revealed that the difference in the percentage of the initial body weight between any 2 groups was not statistically significant (P>0.05).

### Inhibitory effects of TPA and/or DDTC on cell proliferation in PANC-1 tumors

**Figure 6:**
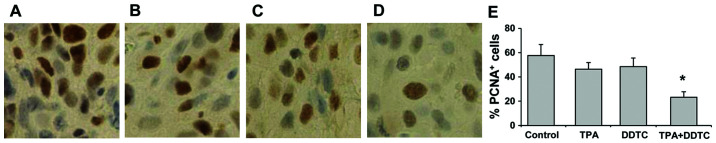
Effect of treatment 12-*O*-tetradecanoylphorbol-13-acetate (TPA) or diethyldithiocarbamate (DDTC) alone or in combination on the expression of proliferating cell nuclear antigen (PCNA) in PANC-1 tumors. Immunohistochemistry with a PCNA antibody was performed on the paraffin-embedded sections of PANC-1 tumors collected from the mice following treatment as described in Fig. 5. Representative micrographs of PCNA immunostaining in tumors from the (A) control, (B) TPA-treated, (C) DDTC-treated and (D) TPA + DDTC-treated groups are shown. PCNA positive cells were determined using a microscope (Nikon Optiphot) and (E) expressed as a percentage of PCNA-positive cells. Each value represents the mean ± SE. ^*^P<0.01, compared to treatment with either agent alone.

The effects of TPA and DDTC on PANC-1 tumor growth were investigated by determining the expression of PCNA in the tumor cells. Immunohistochemistry of PCNA in the paraffin-embedded sections of PANC-1 tumors revealed that treatment of the mice with TPA or DDTC alone reduced the number of PCNA-positive cells in the tumors (Fig. 6B and C). Combined treatment with TPA and DDTC had a more potent inhibitory effect on the number of PCNA-positive cells than treatment with either agent alone (Fig. 6D). The differences in the number of PCNA-positive cells were statistically significant between the group treated with the combination of both agents and the group treated with TPA alone (P<0.01), as well as between the group treated with the combination of both agents and the group treated with DDTC alone (P<0.01; Fig. 6E).

## Discussion

Although previous studies have shown that treatment with TPA or DDTC alone inhibits pancreatic cancer cell growth (13–17,30,31), to the best of our knowledge, the effects and mechanisms of action of these two agents in combination on the growth and the apoptosis of pancreatic cancer cells *in vitro* and *in vivo* have not yet been reported. In the present study, we demonstrated that TPA in combination with DDTC exerted potent growth inhibitory and apoptosis-promoting effects on pancreatic cancer cells. We also demonstrated that the combination of both agents markedly inhibited the growth of PANC-1 xenograft tumors in NCr nude mice. To the best of our knowledge, this is the first study indicating a strong inhibitory effect of the combination of TPA and DDTC on pancreatic cancer cell growth.

In the present study, we determined the effects of TPA and DDTC alone or in combination on PANC-1 cells in 3D cultures. Compared to conventional 2D monolayer cell cultures, the 3D culture system mimics the structural architecture and functional differentiation of tumor tissues (33,34). It is well known that cell-cell and cell-matrix interactions within the 3D microenvironment are important to the physiological function and response of cancer cells to anticancer agents (33,34). In the present study, we found that PANC-1 cells formed a 3D tissue-like morphology in the extracellular matrix Matrigel. Treatment of the PANC-1 cells with TPA and DDTC in combination had a more prominent inhibitory effect on the formation of a tissue-like morphology in the 3D cultures than treatment with either agent alone.

The inhibition of NF-κB activation has been found to enhance the anticancer activities of TPA in leukemia (19) and prostate cancer cells (20). NF-κB is an important cellular regulator of growth and apoptosis. This transcription factor has been connected with multiple aspects of oncogenesis, including the control of apoptosis, the cell cycle, cell differentiation and cell migration (18,35,36). A number of studies have indicated that the activation of NF-κB suppresses cell death pathways and that the activation of NF-κB is required to protect cells from the apoptotic cascade. Chemotherapeutic agents, such as 5-fluorouracil (5-FU), etoposide, docetaxel and gemcitabine have been reported to activate NF-κB in cancer cells (37–40). The activation of NF-κB may be a protective response to treatment with chemotherapeutic agents, while the inhibition of NF-κB has been shown to enhance the anticancer activity of chemotherapeutic agents (37–40). In the present study, luciferase reporter assay revealed that TPA increased NF-κB activity. Treatment with DDTC markedly inhibited NF-κB activity, decreased the expression of Bcl-2 and enhanced the effects of TPA on the PANC-1 cells. These findings indicate that TPA in combination with pharmacological inhibitors of NF-κB may thus be an effective strategy for improving the therapeutic efficacy of TPA in pancreatic cancer.

Previous research by our team demonstrated that the peak blood levels of TPA ± SD value in several patients who received an intravenous (i.v.) infusion of TPA (0.125 mg/m^2^) was 1.75±0.55 ng/ml and ranged between 0.3 and 5.2 ng/ml. The concentrations of TPA used to obtain an inhibitory effect on pancreatic cancer cells in the present study (0.1–1 ng/ml; 0.16–1.6 nM) are clinically achievable (9,41). Concentrations of DDTC used in some previous *in vitro* studies have ranged from nanomolar (nM) to micromolar (*μ*M) and the treatment time has varied between 30 min to 24 h (21,26,44,45). Instead of using a high concentration and a short treatment time, we found that treatment with lower concentrations (50–200 nM) of DDTC for 72 h markedly inhibited the growth and induced the apoptosis of pancreatic cancer cells. The concentrations of DDTC used in the present study were much lower than the blood levels of DDTC in humans (42,43).

A strong inhibitory effect of TPA and DDTC on the growth of PANC-1 xenograft tumors in nude mice was observed in the present study. Treatment of NCr nude mice with i.p. injections of TPA and DDTC in combination more potently inhibited the growth of PANC-1 tumors than treatment with either agent alone. Immunohistochemical analysis revealed that cancer cell growth (proliferation), as reflected by the expression of PCNA, was significantly lower in the tumors from the mice treated with TPA + DDTC than in the tumors from the mice treated with either TPA or DDTC alone. At the doses used in the present study, TPA and DDTC alone or in combination appeared to be non-toxic as no differences in body weight were observed in the animals following treatment. Furthermore, no abnormalities were observed in the major organs at the end of the experiment (data not shown). Further studies are required to establish the plasma levels of TPA and DDTC in relation to their combined inhibitory effect on pancreatic tumors in suitable animal models.

In conclusion, in the present study, we demonstrated that TPA in combination with DDTC markedly inhibited pancreatic cancer cell growth and induced the apoptosis of human pancreatic cancer cells. In addition, we found that treatment of NCr nude mice with a combination of TPA and DDTC inhibited the growth of xenograft PANC-1 tumors. TPA in combination with pharmacological inhibitors of NF-κB, such as DDTC, may thus be an effective approach with which to inhibit the growth of pancreatic cancer.

## Figures and Tables

**Table I:** Effects of TPA and DDTC alone or in combination on the apoptosis of PANC-1 cells.

Treatment	Apoptotic cells (%)	Relative caspase-3 activity
Control	2.1±0.2	1.0
TPA (0.16 nM)	4.0±0.3	1.7±0.2
TPA (0.32 nM)	7.2±0.3	4.1±0.5
DDTC (20 nM)	5.6±0.4	2.7±0.3
DDTC (50 nM)	10.7±1.1	5.2±0.5
TPA (0.16 nM) + DDTC (20 nM)	19.7±2.6^a^	9.8±0.8^a^
TPA (0.32 nM) + DDTC (50 nM)	32.5±2.4^a^	13.0±1.0^a^

PANC-1 cells were seeded at a density of 0.2×10^5^ cells/ml in cell culture dishes and incubated for 24 h. The cells were then treated with TPA and DDTC alone or in combination for 72 h. Apoptotic cells were determined by morphological assessment and by caspase-3 assay. Statistical analysis using ANOVA with Tukey-Kramer multiple comparison test revealed that the differences in the number of apoptotic cells between the group treated with the combination of both agents and the groups treated with either TPA or DDTC alone were statistically significant.

a P<0.001. TPA, 12-*O*-tetradecanoylphorbol-13-acetate; DDTC, diethyldithiocarbamate.
